# Agreement and correlation of abdominal skeletal muscle area measured by CT and MR imaging in cirrhotic patients

**DOI:** 10.1186/s12880-022-00932-0

**Published:** 2022-11-24

**Authors:** Zhengyu Xu, Jia Luo, Dawei Yang, Hui Xu, Jidong Jia, Zhenghan Yang

**Affiliations:** 1 Department of Medical Technology, Shaanxi University of Chinese Medicine, Middle Section of Century Avenue, Xianyang, 712046 Shaanxi China; 2 Department of Radiology, Beijing Friendship Hospital, Capital Medical University, Yongan Road 95, West District, Beijing, 100050 China; 3 Department of Geriatrics, Beijing Friendship Hospital, Capital Medical University, Beijing, 100050 China; 4 Beijing Key Laboratory of Translational Medicine on Liver Cirrhosis, Liver Research Center, Beijing Friendship Hospital, Capital Medical University, Yongan Road 95, West District, Beijing, 100050 China; 5 National Clinical Research Center for Digestive Diseases, Beijing, 100050 China

**Keywords:** Skeletal muscle, Magnetic resonance imaging, Cirrhosis, Sarcopenia

## Abstract

**Background:**

CT-based abdominal skeletal muscle area (SMA) serves as a standard for assessing muscle mass in patients with cirrhosis. Few studies have used MR imaging to measure SMA in cirrhotic patients. The purpose of this study was to investigate the agreement and correlation of the SMA measured by MRI and CT in cirrhotic patients.

**Methods:**

CT and MR images from 38 cirrhotic patients were analyzed using the Slice-O-Matic V5.0 software. One observer independently measured SMA at the mid-third lumbar vertebral (L3) level on CT and MR images. The intraclass correlation coefficient (ICC), Pearson correlation coefficient, and Bland–Altman plot were used to evaluate the agreement and correlation between CT and MRI SMA and their relationship with the sarcopenia severity and Child–Pugh grades.

**Results:**

CT and MRI had a high intraobserver agreement, with ICCs ranging from 0.991 to 0.996. CT and MRI measurements were closely correlated (r = 0.991–0.998, all for *P* < 0.01), and the bias of the measurements was 0.68–3.02%. Among all MR images, T1w water images had the strongest correlation (r = 0.998, *P* < 0.01) and the minimum bias of 0.68%. The measurements of mid-L3 SMA on CT and T1w water images remained highly consistent in cirrhotic patients with different severities of sarcopenia and Child–Pugh grades.

**Conclusions:**

MRI and CT showed high agreement and correlation for measuring mid-L3 SMA in cirrhotic patients. In addition to CT, MR images can also be used to assess muscle mass in cirrhotic patients, regardless of the severity of sarcopenia and Child–Pugh grades.

**Supplementary Information:**

The online version contains supplementary material available at 10.1186/s12880-022-00932-0.

## Background

Sarcopenia has been described as a gradual loss of muscle mass accompanied by a decrease in muscle strength and physical performance [1]. Due to the development of hepatic dysfunction and other systemic diseases secondary to cirrhosis [2], 40–70% of cirrhotic patients have sarcopenia, especially males and those with alcoholic liver disease [3]. In cirrhotic patients, previous studies have confirmed that sarcopenia is a predictor of a high risk of serious infections [4], a high probability of hepatic encephalopathy [5], and lower survival [6]. Therefore, an early and accurate diagnosis of sarcopenia in cirrhotic patients is of critical clinical importance to guide treatment and improve prognosis.

In general, sarcopenia is diagnosed by the combination of muscle mass, strength, and physical performance [7, 8]. However, most of the relevant research related to cirrhosis has followed the latest guidelines [2] to assess sarcopenia using muscle mass in recent years. The skeletal muscle index (SMI) at the third lumbar vertebra (L3) level derived by measuring skeletal muscle area (SMA) at the L3 level on computed tomography (CT) images is now a common method of assessing muscle mass [9]. CT is a common imaging modality that is used to diagnose cirrhosis and assess its complications, such as ascites and portal hypertension. Due to the high accuracy and reliability of SMA measured on CT imaging [10], CT-based SMA at L3 was considered to be the criterion for assessing the muscle mass of cirrhotic patients in the latest American Association for the Study of Liver Diseases (AASLD) guidance [2]. However, considering the presence of radiation exposure from CT examinations and the fact that most cirrhotic patients require regular follow-up, the risk of accumulated radiation exposure from CT examinations cannot be ignored.

Magnetic resonance imaging (MRI) is a non-radiation imaging technique and multiple examinations have no adverse effects on cirrhotic patients. Given its higher soft-tissue resolution and comparable spatial resolution, MRI has more advantages compared to CT for the diagnosis of cirrhosis and the surveillance of hepatocellular carcinoma in the early stage [11, 12]. In addition, the higher soft tissue resolution also facilitates the identification of muscle and adipose tissue and provides a more accurate measurement of SMA. Previous research has observed a high agreement and correlation between SMA measured on MRI and CT. However, most research has been performed on patients with kidney disease rather than liver disease [13, 14]. The study by Sinelnikova et al. [15] evaluated chronic liver disease patients but did not focus on cirrhotic patients and measured SMA based on the first lumbar (L1) level, which did not meet the current diagnostic criteria for sarcopenia. We selected water-fat imaging based on chemical shift encoding to assess muscle mass in this study. This sequence is a regular sequence for abdominal MR examinations and has advantages in the differentiation of muscle and adipose tissue. Hence, we hypothesized that water-fat imaging based on chemical shift encoding has comparable and practical performance in measuring SMA as compared to CT in cirrhotic patients.

Therefore, the purpose of this research is to assess the agreement and correlation between SMA measured on chemical shift encoding-based water-fat imaging and CT in cirrhosis and to investigate whether there are differences in SMA measured on MR and CT imaging in patients with different degrees of sarcopenia and Child–Pugh grades.

## Methods

This prospective research followed the ethical standards of the Declaration of Helsinki, which was authorized by our ethical review committee, and signed informed permission was received from each subject.

### Research population

**Fig. 1:**
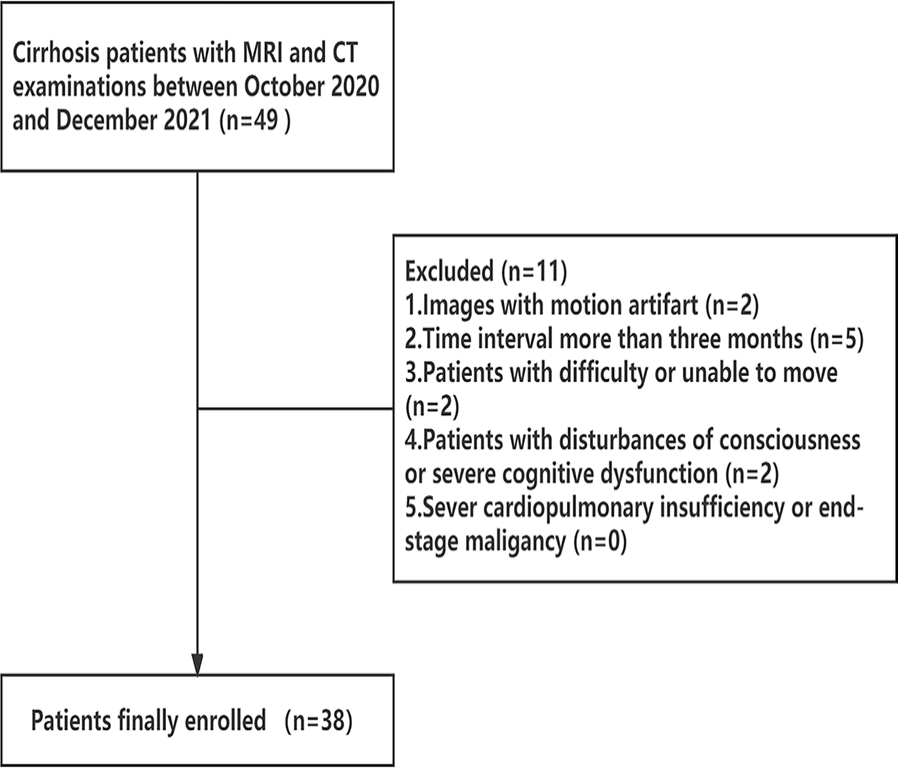
Flowchart of patient inclusion

The research subjects were enrolled in the Department of Radiology between October 2020 and December 2021. The inclusion criteria were as follows: (1) patients who underwent both abdominal CT and MRI scans within a short time gap due to the requirement to examine the portal venous blood flow and liver parenchyma in these patients and (2) patients who met the Chinese Society of Hepatology's diagnostic criteria for liver cirrhosis in 2019 [16]. The exclusion criteria consisted of patients who (1) had images with motion artifacts, (2) had a CT and MRI examination time interval of more than three months, (3) had nervous system diseases or bone and joint diseases that made them unable to exercise, (4) had disturbances of consciousness (in case of overt hepatic encephalopathy) or severe cognitive dysfunction which made them unable to cooperate with the investigation, (5) severe cardiopulmonary insufficiency or end-stage malignancy. Figure 1 shows a flowchart of the patient selection.

### Image acquisition

As a component of the diagnostic workup, the imaging data from the cirrhotic patients who underwent CT and MRI tests were obtained. A 256-row detector CT (Revolution CT, GE Healthcare, Milwaukee, WI, USA) with automatic tube current modulation technology was used to collect CT images from patients with cirrhosis. MR images were obtained from three different 3.0T MRI scanners: Siemens (Magnetom Prisma, Siemens Healthineers, Erlangen, Germany), GE Healthcare (Discovery 750 W, GE, Milwaukee, USA), and Philips (Ingenia, Philips, Best, The Netherlands). The MR imaging protocol consisted of the following sequences: coronal T2-weighted (T2w) single-shot fast spin-echo imaging, transverse T2w fast spin-echo imaging, three-dimensional (3D) gradient-echo T1-weighted (T1w) water-fat separation imaging, axial diffusion-weighted single-shot spin-echo echo-planar imaging, and contrast-enhanced multiphasic MRI. The T1w in-phase, T1w out-of-phase, T1w water, and T1w fat images obtained from breath-hold 3D axial DIXON for Siemens, LAVA-flex for GE, and mDIXON for Philips were used for image analysis. To avoid bias among the different machines, the primary MR imaging parameters were kept consistent in this research. The imaging parameters for MRI and CT are shown in Table 1. The upper border of the CT and MRI scans was the top of the diaphragm and the lower border was the lower edge of the L3 vertebral. For this research, all of the images used for analysis were non-contrast axial images.

**Table 1:** Imaging protocol for MRI and CT

	GE	Siemens	Philips	CT
	LAVA-flex	DIXON	mDIXON	
Tube voltage (kV)	–	–	–	120
Echo time, TE_1_ (ms)	1.10	1.23	1.32	–
TE_2_ (ms)	2.30	2.46	2.40	–
Repetition time, TR (ms)	4.40	3.89	3.70	–
Slice thickness (mm)	4	3	3	5
Field of view, FOV (mm)	380–420	380–420	380–420	300–400
Matrix	288 × 224	288 × 216	252 × 228	512 × 512
Flip angle (°)	15	10	10	–

*TE* Echo time, *TR* Repetition time, *FOV* Field of view

### Image analysis

The mid-L3 segment axial images were selected for the CT and MRI analysis. The three vertebrae caudal to the last thoracic vertebra were counted and cross-referenced to the coronal and sagittal planes to obtain mid-L3 axial CT and MR images, and the slice where both the transverse processes were most prominent was designated as the mid-L3 level. All images were analyzed twice by the radiologist (D.W.Y) with more than 10 years of experience in abdominal imaging using Slice-O-Matic software v5.0 (Tomovision, Magog, Canada). The interval between the two analyses was greater than one month to reduce bias, with the observer blinded to the prior segmentation results. The mean of two measurements was used to analyze the agreement and correlation between CT and MRI.

**Fig. 2:**
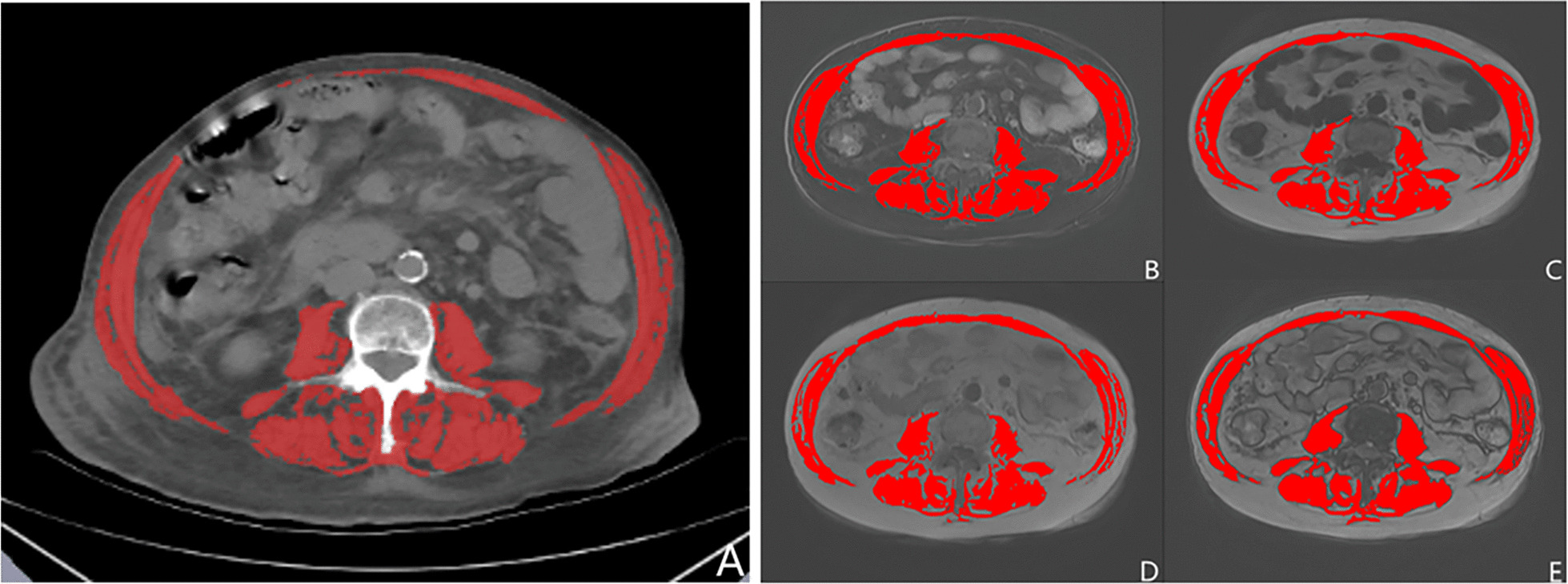
Skeletal muscle area segmentations from CT and MRI. **A** CT images, **B** T1-weighted (T1w) water images, **C** T1w fat images, **D** T1w in-phase images, and **E** T1w out-of-phase images

For CT, the software distinguished between muscle tissue and adipose tissue based on the Hounsfield unit (HU) differences. Our research used a previously widely accepted skeletal muscle threshold (− 29 to + 150) to segment the images (Fig. 2A). For MRI, the semiautomatic “Region-Growing” and “Morpho” were used to segment the images. The histograms described in “Regional Growth” can assist in determining the tissue thresholds on each MRI DICOM. However, the histogram’s first, second, third, and fourth peaks generally indicate air, muscle, bone, and adipose tissue, respectively. Mathematical morphological functions were the basis for the work of the “Morpho”. The MR images were segmented into multiple regions that contained only muscle or adipose tissue. The rectus abdominis, transversus abdominis, paraspinal muscles, quadratus lumborum, psoas major, external oblique, and internal oblique muscles were all included in the SMA measurement. The observer manually outlined the skeletal muscle, by avoiding intramuscular adipose tissue, in an attempt to only extract the area of the skeletal muscle tissue (Fig. 2B–E).

### Reference standard

Cirrhosis and Child–Pugh scores were diagnosed and determined by an experienced gastroenterologist according to the criteria developed by the Chinese Society of Hepatology in 2019 [16]. The values of the SMI at mid-L3 were calculated as follows: SMI (cm^2^/m^2^) = (SMA in cm^2^)/(height in meters^2^). Patients were diagnosed with sarcopenia if the SMI value was < 50 cm^2^/m^2^ for males and < 39 cm^2^/m^2^ for females [9]. The classification of sarcopenia was based on the guidelines of the Asian Working Group for Sarcopenia (AWGS) [8]. Patients were considered to have low grip strength if the grip strength value was < 28 kg for males and < 18 kg for females. Patients were considered to have a low gait speed if the 6-m walk test was < 1 m/s. All patients were further categorized into three subgroups as follows: no sarcopenia (SMI ≥ 50 cm^2^/m^2^ in men, ≥ 39 cm^2^/m^2^ in women), sarcopenia (low SMI and low grip strength or low gait speed), and severe sarcopenia (low SMI, low grip strength, and low gait speed).

### Statistical analysis

SPSS software version 22.0 (SPSS Inc. Chicago, IL) and GraphPad Prism 9 (GraphPad Software Inc. San Diego, CA, USA) were used for statistical analysis. The intraclass correlation coefficient (ICC) was used to assess the intraobserver agreement of the SMA measurements on CT and MR images. The agreement and correlation of the SMA measured on CT and MR images were assessed for all patients and their subgroups with Pearson correlation coefficients and Bland–Altman plots. The statistical significance level was set at *P* < 0.05.

## Results

### Patient characteristics

After excluding 11 patients for various reasons (Fig. 1), 38 patients with cirrhosis were included in our research. The CT and MRI examinations were performed within a period of 2 to 89 days (mean ± SD, 39 ± 39.52 days; median 25 days). Table 2 summarizes the baseline characteristics of the included patients in the research.

**Table 2:** Baseline characteristics

	Total (n = 38)	Male (n = 25)	Female (n = 13)
Age (y), mean ± SD	60 ± 12.6	58 ± 13.2	65 ± 10.0
Height (m), mean ± SD	1.68 ± 0.08	1.71 ± 0.07	1.62 ± 0.04
Grip strength (kg), mean ± SD	28.07 ± 10.69	28.42 ± 10.62	27.94 ± 10.81
6-m walk test (s), mean ± SD	8.21 ± 2.56	7.93 ± 2.51	8.74 ± 2.66
No sarcopenia	10	10	0
Sarcopenia	16	9	7
Severe sarcopenia	12	6	6
Child–Pugh A	12	8	4
Child–Pugh B	18	13	5
Child–Pugh C	8	4	4

### Intraobserver agreement for SMA

The two measurements of SMA by the observer are shown in Table 3. The ICC value for the observer's two SMA assessments on the CT images was 0.996. The intraobserver agreement of the MR images was slightly lower than that of the CT images. The intraobserver ICC values were 0.991, 0.993, 0.994, and 0.991 for the T1w in-phase, T1w out-of-phase, T1w water, and T1w fat images, respectively. Based on these findings, the measurements from CT and MRI showed high intraobserver agreement.

**Table 3:** Intraobserver agreement for SMA measured on CT and MR images

	SMA (cm^2^), mean ± SD			Intraobserver ICC (95% CI)
	The first measurement	The second measurement	Mean	
CT	120.17 ± 34.85	121.24 ± 34.38	120.70 ± 34.58	0.996 (0.991–0.998)
T1w in-phase	117.18 ± 33.28	115.91 ± 33.79	116.55 ± 33.46	0.991 (0.982–0.995)
T1w out-of-phase	117.14 ± 33.78	115.55 ± 34.22	116.35 ± 33.94	0.993 (0.987–0.997)
T1w water	119.48 ± 34.19	117.56 ± 33.63	118.52 ± 33.86	0.994 (0.988–0.997)
T1w fat	117.67 ± 33.80	115.79 ± 35.36	116.73 ± 34.52	0.991 (0.983–0.995)

*SMA* Skeletal muscle area, *ICC* Intraclass correlation coefficient, *T1w* T1-weighted

### Agreement and correlation between SMA measured on MRI and CT images

**Fig. 3:**
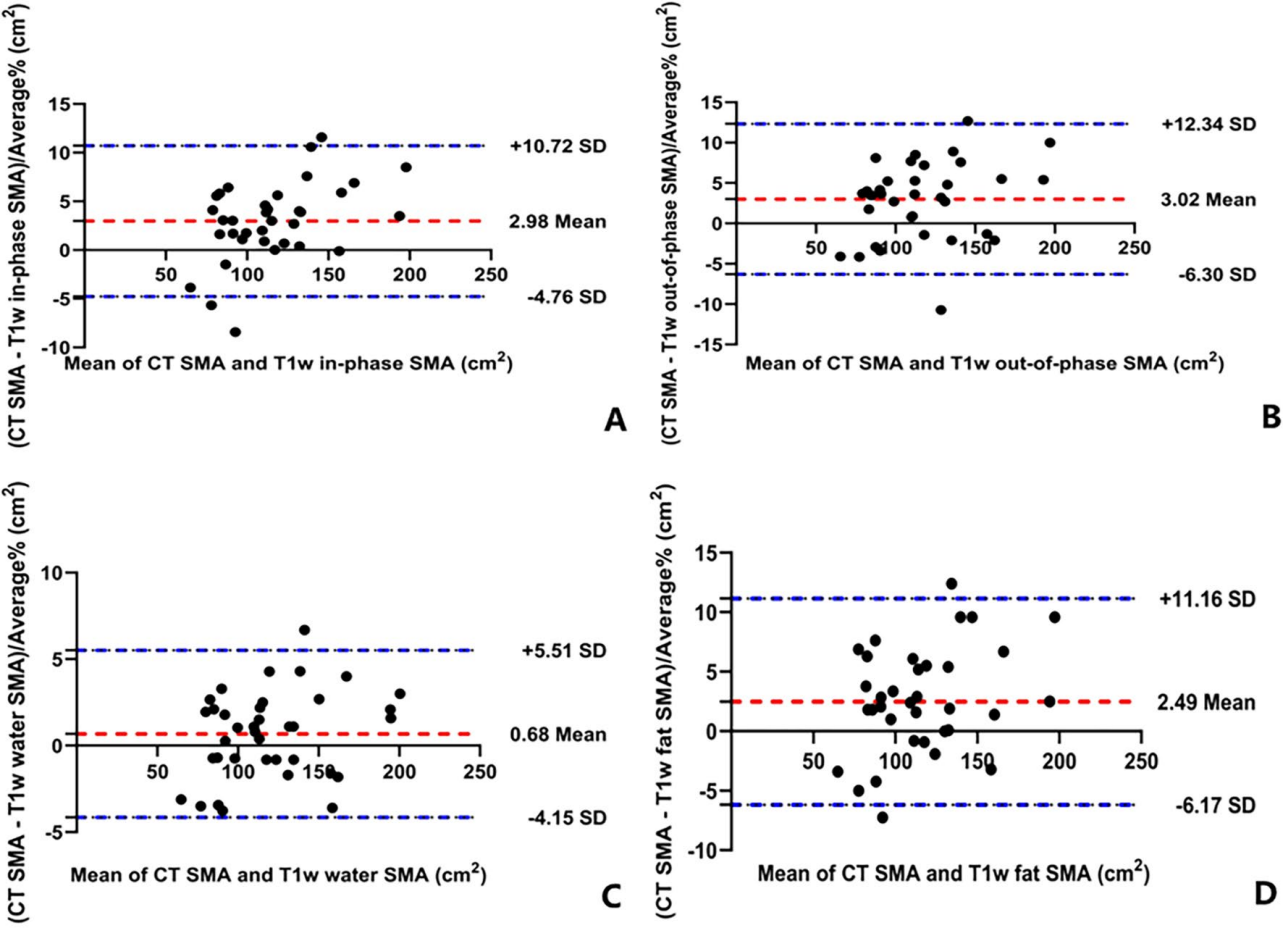
Bland–Altman plots of skeletal muscle area between CT and MRI. **A** CT SMA versus T1-weighted (T1w) in-phase SMA, **B** CT SMA versus T1w out-of-phase SMA, **C** CT SMA versus T1w water SMA, **D** CT SMA versus T1w fat SMA

The SMA presented in Table 3 was obtained from the mean of the two measurements. The correlation between the SMA measured between CT and each MR image was very strong and statistically significant (r = 0.991–0.998, all for *P* < 0.01). The SMA measured on the T1w water images had the most significant association between MRI and CT imaging (r = 0.998, *P* < 0.01). The Bland–Altman plots demonstrated that the CT and MRI methods had a good level of agreement in assessing SMA (Fig. 3). The bias of the MR images to CT is shown in Table 4, and the minimum bias of the T1w water images was 0.68%.

**Table 4:** Consistency and correlation between SMA measured on MRI and CT images

	Pearson correlation coefficient	Bias (%)	LoA (cm^2^)
T1w in-phase versus CT	0.994**	2.98	(− 4.76; 10.72)
T1w out-of-phase versus CT	0.991**	3.02	(− 6.30; 12.34)
T1w water versus CT	0.998**	0.68	(− 4.15; 5.51)
T1w fat versus CT	0.992**	2.49	(− 6.17; 11.16)

*SMA* Skeletal muscle area, *T1w* T1-weighted

**Statistically significant at *P* < 0.001

### T1w water versus CT images agreement in different severity of sarcopenia and Child–Pugh grades

The SMA was significantly different in the patients with different severities of sarcopenia (Table 5). The SMA assessed on the CT and T1w water images in different sarcopenia groups had a substantial positive correlation (r = 0.990–0.996, all for *P* < 0.01), with the group with severe sarcopenia having the strongest connection (r = 0.996, *P* < 0.01). The Bland–Altman analysis showed that there was a slight bias in the CT and MRI measurements among each sarcopenia group (Table 5). In the patients with different Child–Pugh grades, the Pearson’s correlation coefficients of the Child–Pugh A, B, and C groups were 0.999, 0.995, and 0.997, with biases of 1.25%, 0.91%, and − 0.56%, respectively. The SMA measured on CT and MR images showed a high correlation and agreement, regardless of the Child–Pugh grades.

**Table 5:** The SMA measured on MRI and CT images in different Child–Pugh grades and sarcopenia severity

	CT SMA (cm^2^), mean ± SD	T1w water SMA (cm^2^), mean ± SD	Pearson correlation coefficient (T1w water vs CT)	Bias (T1w water vs CT) (%)	LoA (T1w water vs CT) (cm^2^)
No sarcopenia	167.27 ± 22.44	165.53 ± 22.80	0.990**	1.74	(− 4.50; 7.98)
Sarcopenia	106.20 ± 17.04	105.18 ± 16.94	0.994**	1.02	(− 2.49; 4.53)
Severe sarcopenia	99.54 ± 22.64	100.10 ± 22.02	0.996**	− 0.57	(− 4.60; 3.46)
Child–Pugh A	134.74 ± 44.42	133.49 ± 43.42	0.999**	1.25	(− 3.15; 5.66)
Child–Pugh B	109.77 ± 23.21	108.86 ± 22.55	0.995**	0.91	(− 2.54; 4.35)
Child–Pugh C	121.69 ± 36.97	122.25 ± 36.78	0.997**	− 0.56	(− 7.69; 6.56)

*SMA* Skeletal muscle area, *T1w* T1-weighted

**Statistically significant at *P* < 0.001

## Discussion

This research confirmed a relatively high intraobserver agreement between MRI and CT for measuring SMA in cirrhotic patients. These results were consistent with the conclusions of the research conducted by Tandon et al. [17] and Khan et al. [13] in liver transplant donors and patients with kidney disease, respectively. CT imaging has excellent intraobserver agreement as the diagnostic gold standard. The ICC values within the observer for MR imaging were 0.98 and 0.985, respectively [13, 17], which were similar to those reported in this research, with a high intraobserver agreement. Our research did not compare the interobserver agreement between MRI and CT; however, the results of Sinelnikova et al. [15] suggested that MRI and CT had an excellent interobserver agreement, with ICC values of 0.957 and 0. 946, respectively. Therefore, the reproducibility of abdominal SMA segmentation is reliable, regardless of whether MRI or CT is used.

Our research focused on cirrhotic patients, and there was significant agreement and correlation between the MRI and CT measurements. Some previous research has observed similar conclusions, but most of these studies were conducted on patients with kidney disease [13, 14]. Khan et al. [13] and Wang et al. [14] used conventional T2 weighted sequences and IDEAL-IQ sequences, and both studies reported that CT and MR imaging had a high level of agreement and correlation. The outcomes of the two studies were a bias of 0.74% with a Pearson correlation coefficient of 0.997 for Khan et al. [13] and a bias of 2.2% with a Pearson correlation coefficient of 0.995 for Wang et al. [14]. The research by Sinelnikova et al. [15] focused on chronic liver disease patients, with findings consistent with our research. However, all analyses were based on the L1 level instead of the L3 level, which served as a muscle mass assessment criterion. Our research measured SMA in cirrhotic patients on the T1w in-phase, T1w out-of-phase, T1w water, and T1w fat images. The results demonstrated a Pearson correlation coefficient of 0.991–0.998, with a 0.68–3.02% bias. The agreement and correlation between different MR images and CT images were significant, with the T1w water images having the highest agreement and correlation with the measurements of CT images. In addition to CT, MRI can also be applied to the assessment of muscle mass in patients with cirrhosis. Our findings provide a new option for the assessment of muscle mass in cirrhotic patients.

In this study, we chose water-fat imaging based on chemical shift encoding to measure SMA at the mid-L3 level in patients with cirrhosis. As a routine sequence for liver MR examinations, it is especially valuable for diagnosing hepatic steatosis and steatosis within cirrhotic nodules. The sequence distinguishes between water and adipose tissue by the phase shift caused by the difference in the fat–water resonance frequency [18]. The four images were obtained in one breath-hold acquisition with a short scanning time, including in-phase, out-of-phase, water, and fat images. Water–fat imaging based on chemical shift encoding has the advantage of discriminating muscle and adipose tissue. Our research takes advantage of this sequence to provide a more accurate segmentation for SMA.

The CT and MRI measurements showed good agreement and correlation in cirrhotic patients, while the agreement between MRI and CT in patients with different Child–Pugh grades needs to be further investigated. Due to its importance in the classification of liver function and the prognosis of cirrhotic patients [19], the Child–Pugh score is the most commonly used clinical method to assess hepatic function in cirrhotic patients. We compared the agreement and correlation between SMA measured on CT and T1w water images among patients with different Child–Pugh grades in this study. The results showed that MRI and CT measurements were significantly positively correlated (r = 0.995–0.999, *P* < 0.01) and the bias of the measurements was − 0.56 to 1.25%. There was good agreement between MRI and CT, regardless of the Child–Pugh grade of the patients. Moreover, we found that the SMA of the Child–Pugh A, B, and C groups when measured by MRI were 133.49 cm^2^, 108.86 cm^2^, and 122.25 cm^2^, respectively. The SMA did not decrease with increasing Child–Pugh grade in cirrhotic patients. Kang et al. [20] investigated the causes of this association in their study. The primary cause of sarcopenia is related to factors such as malnutrition rather than liver function. The decline in muscle mass was rarely affected by the Child–Pugh grades.

In cirrhotic patients, the presence of sarcopenia typically suggests a poor prognosis [2, 6], and cirrhotic patients are seldom classified in clinical settings according to the degree of sarcopenia. The recently updated European Working Group on Sarcopenia in Older People (EWGSOP2) [7] and AWGS [8] guidelines have further divided sarcopenia into two subgroups: sarcopenia and severe sarcopenia. Several previous studies have demonstrated that sarcopenia is a reliable and independent predictor of death in cirrhotic patients awaiting liver transplantation [21, 22]. The classification of patients according to the severity of sarcopenia will benefit the treatment and prognosis of cirrhotic patients. Hence, this research referred to the latest guidelines [7, 8] to further divide cirrhotic patients into three subgroups: no sarcopenia, sarcopenia, and severe sarcopenia. The results demonstrated a Pearson correlation coefficient of 0.990–0.996 and a bias of − 0.57 to 1.74% between MRI and CT measurements. MRI and CT showed high agreement and correlation for measuring SMA among the three subgroups, regardless of the severity of sarcopenia. Although the latest AASLD guideline for cirrhotic patients with sarcopenia has only recommended CT as the gold standard in assessing muscle mass [2], our research suggests good agreement between MRI and CT, independent of relevant factors. MRI-based measurements of SMA at the L3 level can be used to assess muscle mass in cirrhotic patients. For those patients who require long-term follow-up to assess their prognosis, the use of MRI to measure SMA at the L3 level may be a better option.

There are several limitations of the study. Firstly, the sample size for this study was relatively small. This is related to the fact that our study required patients to undertake both MR and CT examinations in a relatively short time. In previous similar studies, this factor was also the main reason for limiting the sample size [13–15]. A total of 38 subjects were included in the present study. The sample size of our study was significantly larger compared to previous studies, allowing for positive results and conclusions. Second, our research obtained MR images based on different machines. However, the primary imaging parameters in water-fat imaging based on chemical shift encoding remained generally consistent. Third, our analysis was performed by a single independent well-trained observer. A previous study demonstrated that SMA assessed on CT and MRI has significant interobserver agreement [15]. Therefore, it does not affect the reliability of our results. Additionally, the present study only assessed the agreement between CT imaging and chemical shift-encoded moisture imaging; nevertheless, a variety of sequences need to be considered in future research.

## Conclusion

In conclusion, the water-fat imaging based on chemical shift encoding demonstrated high agreement and correlation with CT in assessing SMA at the mid-L3 level in cirrhotic patients. MRI can also be used to assess muscle mass in patients with cirrhosis as well as CT.

## Supplementary Information

**Additional file 1. Table S1.** The original data of the baseline clinical characteristics of the patients.

## Data Availability

The datasets used and analysed during the current study are available from the corresponding author upon reasonable request.

## Abbreviations

SMA: Skeletal muscle area
L3: Third lumbar vertebral
L1: First lumbar vertebral
ICC: Intraclass correlation coefficient
SMI: Skeletal muscle index
CT: Computed tomography
AASLD: American Association for the Study of Liver Diseases
MRI: Magnetic resonance imaging
T1w: T1-weighted
3D: Three-dimensional
T2w: T2-weighted
TE: Echo time
TR: Repetition time
FOV: Field of view
HU: Hounsfield units
AWGS: Asian Working Group for Sarcopenia
EWGSOP2: European Working Group on Sarcopenia in Older People
